# A stem cell-based toolkit to model Angelman syndrome caused by paternal uniparental disomy of chromosome 15

**DOI:** 10.1007/s13577-025-01287-8

**Published:** 2025-09-16

**Authors:** Francisca Cazaux Mateus, João Camões dos Santos, Maria Arez, Evguenia P. Bekman, Simão T. da Rocha

**Affiliations:** 1 Institute for Bioengineering and Biosciences and Department of Bioengineering, Instituto Superior Técnico, University of Lisbon, Lisbon, Portugal; 2 Associate Laboratory i4HB, Institute for Health and Bioeconomy, Lisbon, Portugal; 3 The Egas Moniz Center for Interdisciplinary Research, Caparica, Almada, Portugal

**Keywords:** Angelman syndrome, UBE3A, IPSCs, Disease modeling, Paternal uniparental disomy

## Abstract

Angelman syndrome is a rare neurodevelopmental disorder caused by the loss of function of the maternally inherited *UBE3A* gene within the chr15q11-q13 region. This gene is subjected to a tissue-specific form of genomic imprinting leading to the silencing of the paternal allele in neurons. Angelman syndrome can result from various (epi)genetic mechanisms, with paternal uniparental disomy of chromosome 15 (patUPD15) being one of the rarest and least studied due to the absence of suitable models. To address this gap, we generated three independent induced pluripotent stem cell (iPSC) lines from individuals with Angelman syndrome caused by patUPD15, alongside genetically matched unaffected familial controls. Peripheral blood mononuclear cells (PBMCs) were reprogrammed into iPSCs using a non-integrative Sendai virus-based approach expressing the Yamanaka factors. All iPSC lines underwent rigorous quality control, confirming stem cell identity, trilineage differentiation potential, and genetic and epigenetic integrity. This newly established iPSC toolkit provides a powerful platform to investigate the molecular underpinnings of Angelman syndrome caused by patUPD15, paving the way for future translational research and therapeutic development tailored for this understudied form of the disorder.

**Supplementary Information:**

The online version contains supplementary material available at 10.1007/s13577-025-01287-8.

## Introduction

**Fig. 1:**
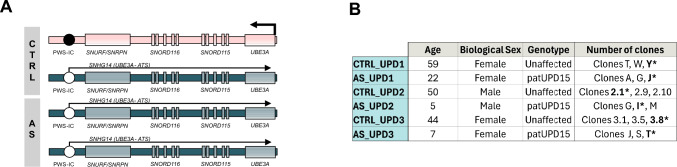
Summary of the newly generated induced pluripotent stem cell lines to model Angelman syndrome caused by paternal uniparental disomy for chromosome 15. **A** Schematic representation of the distal portion of the chr15q11-q13 imprinted region displaying the epigenetic and transcriptional profile in neurons of the unaffected control (CTRL) and paternal uniparental disomy for chromosome 15 (AS) individuals. The maternal chromosome is marked in pink and the paternal chromosome is marked in blue. Arrows represent the expression of a given gene. White circle, unmethylated Prader–Willi Syndrome-Imprinting Center (PWS-IC); black circle, methylated PWS-IC. The scheme is not drawn to scale. **B** Summary table of induced pluripotent stem cell (iPSC) lines generated from six individuals across three families, each including one unaffected (CTRL_UPD) and one Angelman syndrome (AS_UPD) individual with paternal uniparental disomy of chromosome 15 (patUPD15). Besides the genotype and the number of independent clones generated per individual, the table also contains information on the age and biological sex of each individual. Clones with the * are the ones that were thoroughly characterized in this study

Angelman syndrome is a rare neurodevelopmental disorder with no cure, characterized by severe developmental delay, speech impairment, ataxia, seizures and a happy, excitable demeanor [1]. This disorder is caused by the loss of function of the maternally inherited copy of the *UBE3A* gene on the 15q11-q13 chromosomal region [2, 3]. This region is regulated by genomic imprinting, an epigenetic mechanism that controls parent-of-origin-specific gene expression [4]. In the case of *UBE3A*, its parent-specific expression is only seen in neurons, where the maternal allele is active, while the paternal allele is silenced by the *UBE3A* antisense transcript (*UBE3A-ATS*) (Fig. 1A) [5–8]. In Angelman syndrome, this tissue-specific form of imprinting prevents paternal *UBE3A* from compensating for maternal loss of this gene causing the disease. Loss of maternal *UBE3A* results mainly from four distinct molecular causes [9, 10]: (1) the most prevalent and severe are 5–6 Mb deletions of the maternal chr15q11-q13 (~ 70–80%)—in addition to the loss of *UBE3A* expression, multiple non-imprinted genes in this region, such as the GABA_A_ receptor subunit genes *GABRB3, GABRA5,* and *GABRG3,* are hemizygous and likely haploinsufficient, potentially worsening disease severity; (2) mutations in the maternal copy of *UBE3A* (~ 10–20%)—with no further genetic alteration associated with the disease; (3) imprinting defects (~ 3–5%) due to loss of methylation at the maternal imprinting control region of the chr15q11-q13 region known as Prader-Willi Syndrome-Imprinting Center (PWS-IC)—this region is named after Prader-Willi syndrome, another imprinting disorder caused by distinct genetic defects in the same locus; (4) paternal uniparental disomy for chr15 (patUPD15) (~ 3–5%)—in the two latest cases, *UBE3A* loss of function is accompanied by abnormal biallelic expression of several paternally expressed imprinted genes (*MKRN3, MAGEL2, NDN, SNRPN/SNURF, SNORD116, SNORD115*); this distinct transcriptional profile at chr15q11-q13 makes these Angelman cases unique, though the significance of these expression changes remains unclear.

Most of our understanding of Angelman syndrome pathophysiology comes from the maternal *UBE3A* knockout mouse models, which has been instrumental in uncovering key molecular and neurological features of the disorder [12, 28]. However, while these models replicate some patient symptoms, they are significantly milder and fail to represent Angelman cases caused by megadeletions, patUPD15, or imprinting defects, limiting its clinical and translational relevance. An early Angelman syndrome mouse model created by Cattanach et al. [11] was the closer representation to patUPD15, as it carried a paternal duplication of the homologous chr15q11-q13 region on mouse chromosome 7. While these mice exhibited Angelman-like traits, their use was limited due to complex breeding requirements, preventing widespread application in studying disease mechanisms [11].

To address these limitations, innovative human stem cell-based models have recently emerged to study Angelman syndrome in vitro [16]*.* These include embryonic stem cells (ESCs) genetically engineered to replicate patient-specific mutations and induced pluripotent stem cells (iPSCs), which are directly derived from patients and retain the original molecular cause of the disease. These cells offer a robust model for Angelman syndrome, as they can be differentiated into neurons or brain organoids to study disease mechanisms in a human-relevant context and explore potential therapeutic strategies.

The current collection of Angelman iPSCs covers most of the (epi)genotypes of the disease, but the number of cell lines is scarce and lacks proper isogenic or genetically matched controls [16]. For instance, to our knowledge, only one patUPD15 iPSC line has been reported [17] with no matched healthy control. These limitations significantly undermine the robustness of neuronal models derived from these cell lines, as genetic background variability, together with inherent epigenetic heterogeneity of iPSC models, affects cellular differentiation and functionality [18]. Here, we present a new stem cell tool kit to model Angelman syndrome caused by patUPD15. By reprogramming patient-derived alongside genetically matched familial control somatic cells, we generated and rigorously characterized three independent iPSC pairs. Comprehensive quality controls confirmed this novel resource as a robust and physiologically relevant platform to investigate this understudied cause of Angelman syndrome, paving the way for advanced disease modeling and therapeutic exploration.

## Materials and methods

### Participant recruitment and ethics

The recruitment of the three families was conducted in partnership with the Portuguese Angelman syndrome Association (ANGEL). Blood sample collection from patients and their parents was performed by Germano de Sousa Laboratory Medicine Center under the coordination of the Instituto de Medicina Molecular Biobank (Biobank-iMM). All procedures involving the collection, manipulation, and storage of human cell samples were carried out with prior informed consent from the donors or their legal representatives. These procedures followed the ethical guidelines outlined in European and National regulations (Law 12/2005), with approval from the Ethics Committee of the Lisbon Academic Medical Center (300/22).

Prior to the generation of iPSC lines, confirmation of the genetic diagnosis of patUPD15 was carried out by Unilabs (GENDIA—Genetic Diagnostic Network, Belgium) through a PCR multiplex analysis of 10 specific polymorphic genetic markers in chromosome 15. The analysis showed that all alleles for these markers were homozygous and of paternal origin, with the complete absence of a maternal allele. These findings are consistent with a patUPD15 genotype of isodisomy origin.

### Generation and maintenance of iPSC lines

Peripheral blood mononuclear cells (PBMCs) were isolated from whole blood using Ficoll–Paque and preserved in 20% DMSO (Merck, #D8418) in fetal bovine serum (FBS) (Life Technologies, #10500), following the standardized procedure of Biobanco-iMM. Prior to reprogramming, thawed PBMCs were cultured for 4 days in StemPro™ Medium (Thermo Fisher Scientific, #10639011) supplemented with cytokines: IL-3, IL-6, FLT-3, and SCF (Prepotech/Tebu-bio, #200.03, #200.06, #300-19, #300-07). Subsequently, 5 × 10⁶ cells were transduced using the CytoTune™-iPS 2.0 Sendai Reprogramming Kit (Thermo Fisher Scientific, #A16517), applying the following multiplicity of infection (MOI) for each virus: KOS MOI = 4, *hc-Myc* MOI = 4, and *hKlf4* MOI = 2.4. Three days post-transduction, cells were reseeded onto Matrigel^®^-coated (Corning, #35430) 6-well culture plates and transitioned to mTeSR™ Plus Medium (STEMCELL Technologies, #100-0276) over the following days.

Colonies exhibiting distinctive stem cell morphology were individually picked and transferred to new Matrigel^®^-coated plates in mTeSR™ Plus Medium around 10–15 days post-transduction. For the first five passages, colonies were manually picked and transferred to fresh Matrigel^®^-coated plates in mTeSR™ Plus Medium. Subsequent passages were performed when colonies reached approximately 80% confluency, using 0.5 mM EDTA dissociation buffer (Thermo Fisher Scientific, #15575020), at a 1:3 to 1:6 ratio every 3–4 days. Passaging continued until the absence of the Sendai Virus (SeV) RNA genome was confirmed via RT-qPCR (see below). Once the cells were SeV-free, a biobank was established with three different clones from each donor. iPSCs derived from AS patient cell lines (AS_UPD) and familial controls (CTRL_UPD) were maintained at 37 °C in a humidified incubator with 5% CO₂ and 20% O₂. For cryopreservation, cells were dissociated using 0.5 mM EDTA dissociation buffer and resuspended in a freezing medium consisting of 90% KnockOut™ Serum Replacement (Thermo Fisher Scientific, # 10828028) and 10% dimethyl sulfoxide (DMSO) (Sigma, #D2438). The cell suspension was transferred to cryovials and gradually cooled at a rate of approximately − 1 °C per minute using a CoolCell^®^ LX Cell Freezing Container (Corning, #200-0645). After 24 h, the vials were transferred to liquid nitrogen, for long-term storage.

### Reverse transcription-quantitative polymerase chain reaction (RT-qPCR)

Total RNA was extracted using NZYol (NZYTech^®^, MB18501) from each iPSC clone, as well as from all clones at the final time point of the trilineage differentiation assay. The extracted RNA was then treated with DNase I (Roche^®^, 04716728001) to remove genomic DNA contamination. cDNA synthesis was performed using the Transcriptor High Fidelity cDNA Synthesis Kit (Roche^®^, 5081963001). RT-qPCR was carried out using gene-specific primers (Table S1) and the NZYSupreme qPCR Green Master Mix (2x), ROX Plus (NZYTech, MB44001). Reactions were run in triplicate for 40 cycles on a StepOnePlus real-time PCR system (Applied Biosystems). Gene expression levels were calculated as the fold change of the target gene normalised against the *GAPDH* housekeeping gene (2^–Δ^Ct).

### Mycoplasma detection

iPSC cultures were routinely screened for mycoplasma contamination using the qPCR Mycoplasma Test (Mycoplasmacheck, Eurofins Genomics), following the manufacturer’s instructions.

### Immunofluorescence (IF)

For IF, iPSCs were plated on Matrigel-coated dishes with glass coverslips. Once they reached 70% confluency, cells were fixed with 3% paraformaldehyde (PFA) (Sigma-Aldrich, #158127) [in phosphate-buffered saline (PBS) (Thermo Fisher Scientific, #21600-044)] for 10 min at room temperature. To prevent nonspecific staining, cells were treated for 10 min with 0.1 M glycine in PBS (Sigma-Aldrich, #G8898). Permeabilization was performed using 0.5% Triton X-100 in PBS (Sigma-Aldrich, #T9284) for 4 min on ice. Following permeabilization, cells were blocked with 1% bovine serum albumin (BSA) (Sigma-Aldrich, #A9418) in PBS for at least 15 min at room temperature. Cells were then incubated with specific primary antibodies (Table S2) diluted in 1% BSA for 1 h at room temperature, followed by incubation with secondary conjugated antibodies (Table S2) diluted in 1% BSA for 45 min at room temperature. Nuclei were counterstained with DAPI (0.2 mg/ml; Cat# D9542, Sigma). Images were acquired using a Zeiss Cell Observer microscope.

### Flow cytometry

For flow cytometry analysis, iPSCs from each clone were collected using Accutase (Sigma-Aldrich, A6964) and fixed in 2% PFA in PBS for 15 min at room temperature. The cells were then stained with surface antibodies (SSEA4 or TRA-1–60) (Table S2) diluted in 10% FBS in PBS for 15 min at room temperature. Before analysis on the FACS Canto flow cytometer (BD Biosciences), cells were resuspended in 10% FBS in PBS and transferred to round-bottom tubes. For each experimental sample, at least 10,000 events were recorded within a defined gate based on side scatter (SSC) and forward scatter (FSC). Data were analyzed using FlowJo software V10.

### Trilineage differentiation

The trilineage differentiation potential of iPSCs into endoderm, mesoderm, and ectoderm was assessed using the STEMdiff Trilineage Differentiation Kit (STEMCELL Technologies, #05230) for 5 days (endoderm and mesoderm) and 7 days (ectoderm) following the manufacturer’s instructions. Lineage-specific markers were evaluated through IF and RT-qPCR (Table S1).

### Short tandem repeat (STR) analysis

To verify cell line clonality, DNA extracted from iPSCs and PBMCs was submitted to Genomed SA (Lisbon, Portugal) for STR profiling. The analysis was performed using the AmpFLSTR^®^ Identifiler^®^ Plus PCR Amplification Kit, which enables multiplex PCR amplification of fifteen STR loci (D8S1179, D21S11, D7S820, CSF1PO, D3S1358, TH01, D13S317, D16S539, D2S1338, D19S433, vWA, TPOX, D18S51, D5S818, and FGA) along with the Amelogenin marker for gender determination.

### G-Banding karyotyping

For G-banding karyotyping, iPSCs in the exponential growth phase were treated with colcemid (10 μg/ml; Thermo Fisher Scientific, #15212012) for 5 h at 37 °C to arrest cells in metaphase. Cells were then harvested using Accutase for 7 min at 37 °C and treated with a hypotonic potassium chloride solution for 30 min at 37 °C. Finally, the cells were fixed in a 1:3 (v/v) glacial acetic acid:methanol solution. Karyotype analysis was performed by Genomed SA (Lisbon, Portugal) with chromosomes being analyzed at a 300-to-500-band resolution. The number of metaphase spreads with correct karyotype recorded per iPSC line is mentioned in the figure legend.

### hPSC qPCR genetic analysis

Genomic DNA from each iPSC line was isolated using the Quick-DNA Plus kit (Zymo Research, #D4068). qPCR reactions were prepared following the hPSC Genetic Analysis Kit protocol (STEMCELL Technologies, #07550) and run on a StepOnePlus real-time PCR system (Applied Biosystems). The kit targets common copy number variations in human pluripotent stem cells, including gains in 1q, 8q, 10p, 12p, 17q, 20q11.21, and Xp, and losses in 18q. Data analysis was performed using the kit’s reference-normalized ΔΔCt method, with thresholds for abnormality detection set according to the manufacturer’s guidelines.

### COBRA

Genomic DNA from each cell line was isolated using the phenol:chloroform:isoamyl alcohol (Thermo Fisher Scientific, #15593031) extraction method. A total of 500 ng of genomic DNA was bisulfite-converted using the EZ DNA Methylation-Gold Kit (Zymo Research, # D5006) following the manufacturer’s instructions. The bisulfite-treated DNA was then amplified through nested PCR using specific primers for the PWS-IC region (Table S1) and a high-fidelity PCR enzyme, KAPA HiFi HotStart Uracil+ ReadyMix (Roche®, #KK2801). Finally, the amplified DNA was digested with the *Bsh1236*I (*BstU*I) fast-digest restriction enzyme (Thermo Fisher Scientific, #FD0924), which recognizes CG^CG sites—corresponding to the methylated cytosines in the original DNA. Results were analysed by agarose gel electrophoresis.

## Results

### Clinical information

In collaboration with the Portuguese Association of Angelman syndrome (ANGEL), we recruited twelve families willing to donate blood samples from individuals with Angelman syndrome and a biologically sex-matched parent to generate iPSCs for research and therapeutic advancement. Out of these, three families had children previously diagnosed with Angelman syndrome caused by isodisomic patUPD15, two females and one male, with ages ranging from 5 to 22 years old (Fig. 1B). Due to the limited models available for studying patUPD15 cases of Angelman syndrome, we prioritized generating iPSCs from these individuals and their sex-matched parents. We chose sex-matched parental controls to minimize genetic and epigenetic variability linked to two X chromosomes and X-chromosome inactivation erosion in female iPSCs [19], while accounting for sex-specific phenotypic differences. Importantly, we confirmed the isodisomic patUPD15 molecular diagnosis through PCR multiplex analysis of 10 specific polymorphic markers on chromosome 15 before reprogramming (see “Materials and methods”).

### Establishment of three genetically matched pairs of induced pluripotent stem cells to model Angelman syndrome caused by patUPD15

**Fig. 2:**
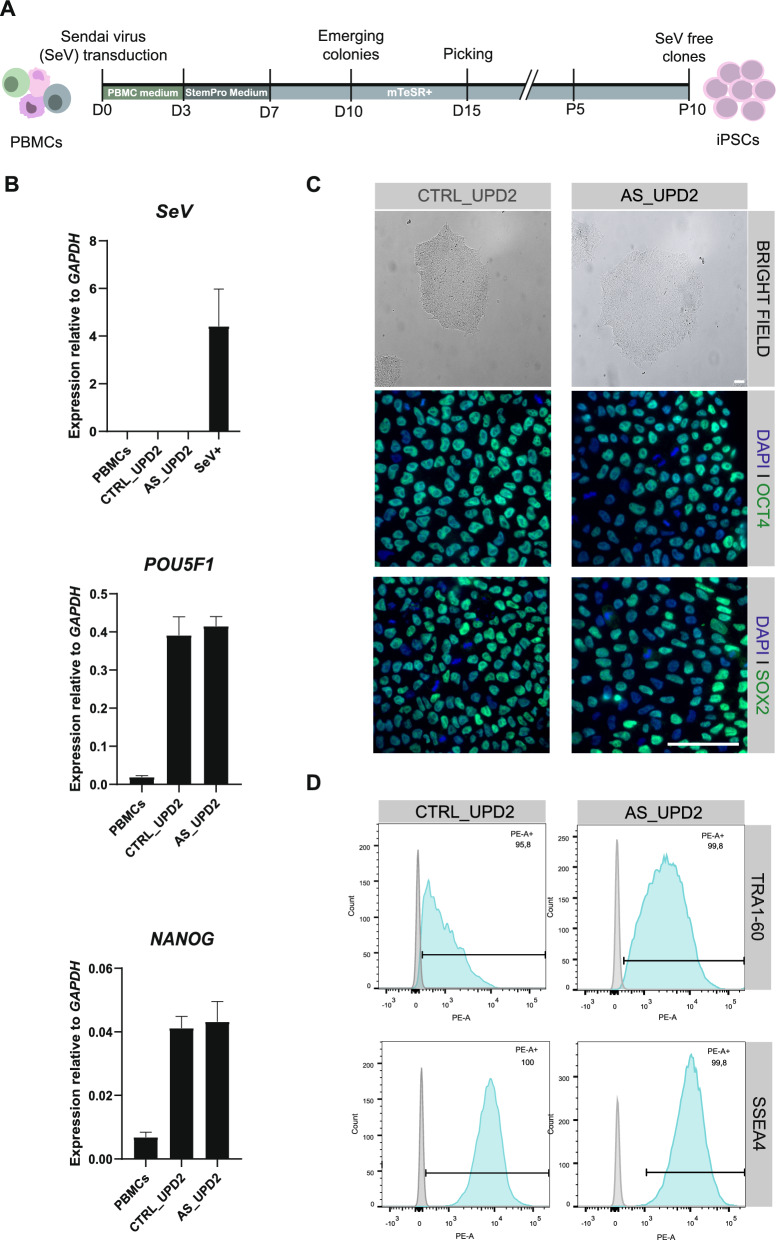
Generation and characterization of stemness markers of newly generated CTRL-UPD2 and AS-UPD2 induced pluripotent stem cell lines. **A** Schematic representation of the non-integrative Sendai Virus (SeV)-based approach to reprogram Peripheral Blood Mononuclear Cells (PBMCs) into induced pluripotent stem cells (iPSCs); For medium formulations (PBMC medium, StemPro medium, mTeSR+), please see “Materials and methods”; the scheme is not drawn to scale in terms of time. **B** Expression levels of *SeV*, *POU5F1* and *NANOG* as measured by Reverse Transcription-quantitative Polymerase Chain Reaction (RT-qPCR) normalized for *GAPDH* housekeeping gene, barplots show mean ± standard deviation (SD); On top, barplot shows *SeV* expression levels in PBMCs, CTRL_UPD2 and AS_UPD2 iPSC lines, as well as infected samples at day 3 of reprogramming (SeV+); In the middle and below, the barplots show expression levels of *POU5F1* and *NANOG*, respectively, in PBMCs, CTRL_UPD2 and AS_UPD2 iPSC lines; *n* = 1 for all samples. **C** On top, bright field image of iPSC colonies for both CTRL_UPD2 and AS_UPD2 iPSC lines; in the bottom, immunofluorescence images of protein expression of stemness nuclear markers OCT4 and SOX2 in green, counterstained by DAPI in blue for both CTRL_UPD2 and AS_UPD2 iPSC lines; scale bar: 50 µm. **D** Quantitative expression of the stemness membrane markers, TRA1-60 and SSEA4, in CTRL_UPD2 and AS_UPD2 iPSC lines by flow cytometry

PBMCs isolated from blood samples of the donors were reprogrammed into iPSCs across three separate reprogramming rounds. In each round, a pair consisting of an Angelman individual and a familial control was reprogrammed side by side. We generated iPSCs using a non-integrative SeV-based method, which transiently overexpresses the Yamanaka factors (*OCT3/4*, *SOX2*, *KLF4*, and *c-MYC*) to reprogram somatic cells. PBMCs were initially cultured for 4 days before being transduced with the SeV carrying the Yamanaka factors. Three days after transduction, cells were gradually transitioned to mTeSR Plus medium and around day ten, stem cell-like colonies began to emerge. Multiple colonies were individually picked as separate clones between days 10–15 after induction. These clones started to be monitored for SeV clearance by RT-qPCR from passage 5. Most of the clones became SeV-negative between passages 5 and 8 (Fig. 2B; Fig. S1A, S2B and data not shown). At least three SeV-negative clones were generated from each individual. All clones were verified for stem cell marker expression (*POU5F1* and *NANOG*) and biobanked in liquid nitrogen.

### Stemness, pluripotency and identity of newly generated induced pluripotent stem cells

Following biobanking, we chose one clonal cell line per individual and submitted to a strict quality control pipeline. After confirming SeV clearance by RT-qPCR, the new cell lines were evaluated for the expression of stemness markers. We confirmed the expression of *POU5F1* (OCT4) and *NANOG* genes by RT-qPCR (Fig. 2B; Fig. S1A, S2A) and OCT4, SOX2 or TRA-1–60 proteins by IF (Fig. 2C; Fig. S1B, S2B). In addition, flow cytometry analysis showed that over 95% of cells were positive for the membrane markers TRA-1–60 and SSEA4 (Fig. 2D; Fig. S1D, S2D).

**Fig. 3:**
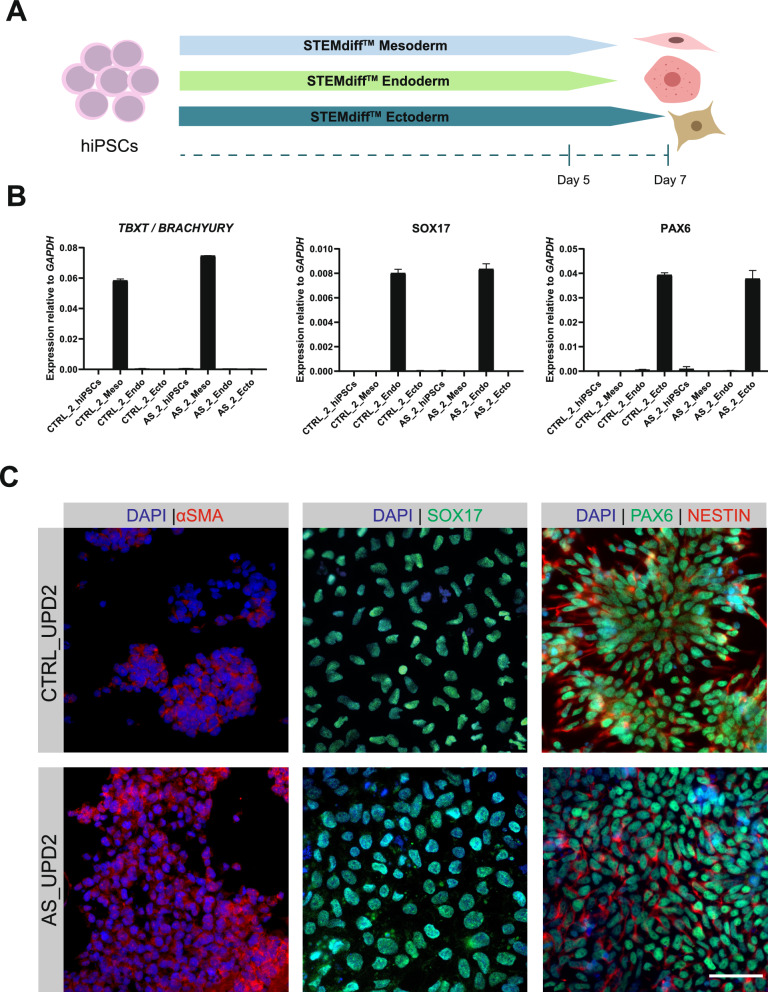
Trilineage differentiation competency of newly generated CTRL-UPD2 and AS-UPD2 induced pluripotent stem cell lines. **A** Schematic representation of the trilineage differentiation protocol to generate the three germline layers, mesoderm, endoderm and ectoderm. **B** Expression levels of *TBXT*/*BRACHYURY*, *SOX17* and *PAX6* markers as measured by Reverse Transcription-quantitative Polymerase Chain Reaction (RT-qPCR) normalized for *GAPDH* housekeeping gene in CTRL_UPD2 and AS_UPD2 induced pluripotent stem cells (iPSC) lines in the undifferentiated state and differentiated down to the mesoderm, endoderm and ectoderm lineages; *n* = 1 for all samples; barplots show mean ± SD of technical replicates. **C** Representative immunofluorescence images of the mesoderm marker (ɑSMA, in red), the endoderm marker (SOX17, in green) and ectoderm markers (PAX6 in green and NESTIN in red) counterstained with DAPI (in blue) in CTRL_UPD2 and AS_UPD2 iPSCs, respectively, differentiated into mesoderm, endoderm and ectoderm; scale bar: 50 µm

After confirming expression of markers of the pluripotent stem cell identity, we proceeded with a trilineage differentiation assay to determine whether these cells commit to the three germ layers. For that, we used a STEMdiff Trilineage Differentiation Kit and evaluated the expression of lineage-specific genes by RT-qPCR and/or IF. Expression of the lineage-specific markers was detected exclusively in their corresponding lineages: *TBXT (BRACHYURY)* was expressed only in the mesoderm, *SOX17* only in the endoderm, and *PAX6* only in the ectoderm for all six cell lines (Fig. 3B; Fig. S3A). Furthermore, IF analysis in the CTRL-UPD2 and AS-UPD2 iPSC lines confirmed the presence of lineage-specific genes at the protein level (PAX6 and NESTIN for ectoderm, αSMA for mesoderm and SOX17 for endoderm), further validating the trilineage differentiation potential of these cells (Fig. 3C).

Next, we performed STR analysis to confirm the identity and clonality of the newly generated iPSC lines. The STR profiles obtained from each iPSC line match exactly with those of their respective donor PBMCs, confirming that the reprogrammed iPSCs were derived from the intended individuals (Table S3).

In summary, all six iPSC lines express key stemness markers, exhibit trilineage differentiation potential, and STR profiles matching the respective donor PBMCs, demonstrating key characteristics typical of pluripotent stem cells.

### Genetic and epigenetic fidelity of newly generated induced pluripotent stem cells

Imprinting and genetic defects are a well-known concern in iPSC generation [20–23]. To address this, we assessed the genetic and epigenetic fidelity of each cell line to detect potential defects arising during reprogramming. Specifically, we analyzed karyotype integrity and the methylation status of the PWS-IC, the key regulatory region controlling imprinting at 15q11-q13. Besides detecting chromosomal abnormalities due to reprogramming/stem cell culture, karyotyping is crucial to determine whether patUPD15 arises from two intact paternal copies of chromosome 15 or from karyotypic abnormalities like Robertsonian translocations. Meanwhile, PWS-IC methylation analysis is expected to distinguish patUPD15 cases from healthy controls by assessing the presence of paternal and maternal epigenotypes, while helping to verify that no imprinting instability occurred at this locus during reprogramming [22].

**Fig. 4:**
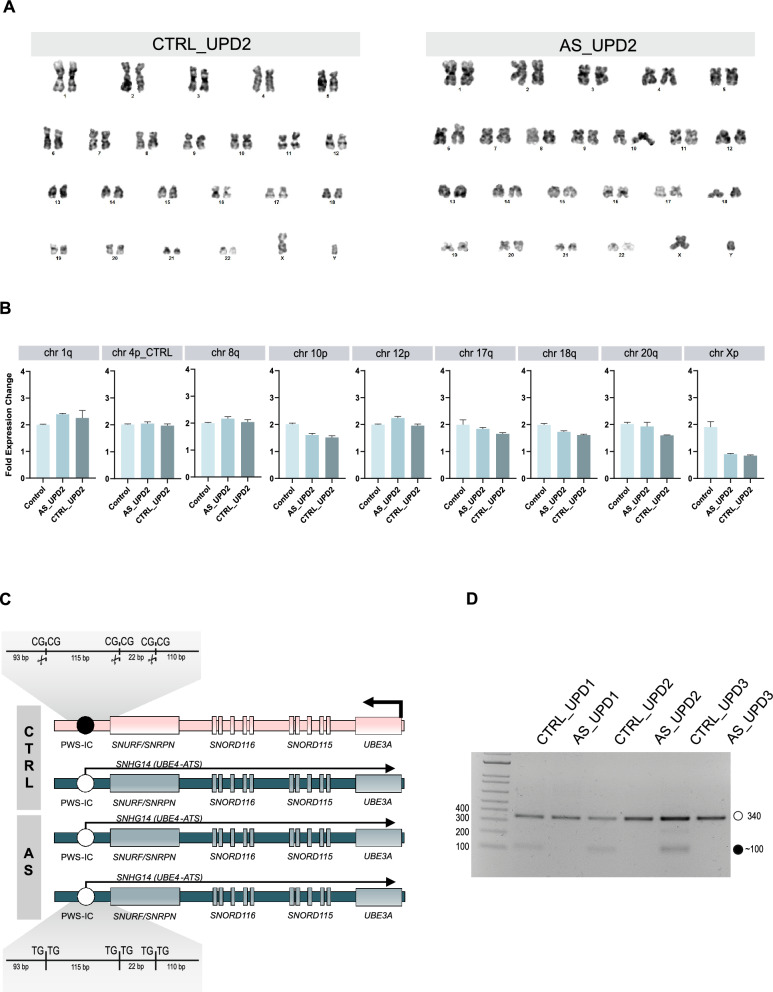
Chromosomal/genetic integrity and methylation analysis of the Prader-Willi Syndrome Imprinting Center in CTRL_UPD2 and AS_UPD2 induced pluripotent stem cells. **A** Representative G-banding karyotype images of CTRL_UPD2 (46,XY, 10 metaphase spreads counted) and AS_UPD2 (46,XY, 27 metaphase spreads counted); 300–500 band resolution. **B** Copy number variations (CNVs) in specific chromosomal regions (1q, 8q, 10p, 12p, 17q, 20q11.21, Xp, and 18q) were determined by a qPCR-based genetic assay to screen for recurrent chromosomal abnormalities in iPSCs. Barplots show mean ± SD. Statistical analysis was performed using one-way ANOVA with a pairwise test and no statistically significant abnormalities were detected below *p*-value of 0.05. **C** Schematic representation of the COmbined Bisulfite Restriction Analysis (COBRA) to evaluate methylation status at the Prader-Willi Syndrome-Imprinting Center (PWS-IC) in controls (CTRL) and patUPD15 individuals (AS); Inset of the PWS-IC region display the DNA with the restriction enzyme recognition sequence upon bisulfite treatment of the methylated maternal and unmethylated paternal alleles; the presence of scissors indicates when the restriction digestion occurs with the expected band sizes in base pairs indicated. **D** Agarose gel image displaying the PWS-IC COBRA analysis for unaffected controls (CTRL_UPD1, CTRL_UPD2, CTRL_UPD3) and the patUPD15 cell lines (AS_UPD1, AS_UPD2, AS_UPD3); white circle, unmethylated band; black circle, methylated band; ladder on the first row is 1 Kb ladder with the size of the smaller bands indicated in **C**

All cell lines displayed a normal karyotype consistent with biological sex, as confirmed by G-band karyotyping, with no signs of karyotypic abnormalities (Fig. 4A; Fig. S4A). In the cases of patUPD15, we confirmed the presence of two intact paternal chromosome 15 copies. Additionally, we applied a qPCR-based genetic assay to screen for recurrent chromosomal abnormalities in iPSCs. specifically gains in 1q, 8q, 10p, 12p, 17q, 20q11.21, and Xp, and losses in 18q, that are not detectable by conventional karyotyping. No statistically significant alterations were identified in any of the six iPSC lines (Fig. 4B; Fig. S4B). Only minor deviations were observed (*e.g.*, chr1q and chr10p in AS_UPD2 or chr10p, chr18q and chr20q in CTRL_UPD2), which may reflect either technical variability or the early emergence of low-level mosaicism (discussed in the Conclusion section).

We also assessed the methylation status of the PWS-IC using a Combined Bisulfite Restriction Analysis (COBRA). This method combines bisulfite treatment of genomic DNA with a restriction digestion allowing to distinguish methylated from unmethylated DNA. Briefly, bisulfite treatment converts unmethylated cytosines to thymines, while methylated cytosines remain unchanged, effectively translating a methylation status to changes in the DNA sequence. Following bisulfite conversion, a restriction assay is performed where only the unaltered DNA sites, with the original cytosines, are digested, distinguishing between methylated and unmethylated imprinting centers (Fig. 4C). Digestion results show the expected restriction patterns identifying the presence of the methylated and unmethylated PWS-IC in the control samples, but only the unmethylated PWS-IC in the Angelman samples (Fig. 4D). These results also confirm that the reprogramming method used to generate these cells did not alter methylation at this specific imprinting center, ensuring that these cells accurately represent the in vitro epigenetic state of individuals with Angelman syndrome and healthy controls.

In conclusion, we generated three pairs of patUPD15 Angelman syndrome iPSC lines and their respective familial controls, demonstrating their stemness and pluripotency, correct karyotype, as well as proper imprinting status at the 15q11-q13 locus.

## Conclusion

We successfully generated and characterized three independent pairs of iPSCs from Angelman individuals with patUPD15 and their corresponding familial controls. This iPSC toolkit provides a powerful platform for studying patUPD15-specific disease mechanisms, expanding patient-derived models for this molecular cause while minimizing genetic background variability. With the ability of iPSCs to differentiate into neuronal lineages and brain organoids, this toolkit offers significant potential for studying the neurodevelopmental and molecular consequences of Angelman syndrome caused by patUPD15 and for developing drug and therapy screening strategies specifically tailored to these cases.

These iPSCs exhibit robust pluripotency, demonstrated by the expression of undifferentiated state markers and their ability to differentiate into the three germ layers, and possess a normal karyotype and correct imprinting status at the PWS-IC, ensuring their suitability for disease modeling. Although no strong evidence of recurrent mutations was detected, low-level gains or losses could still be present in a small subset of cells within an hPSC line. Such changes should not be overlooked, and the genetic (and epigenetic) integrity of these and other iPSC lines should be routinely monitored, for example, every five passages [18].

Modeling patUPD15 cases of Angelman syndrome is challenging due to the lack of an accessible mouse model and the difficulty of generating CRISPR/Cas9-edited isogenic systems. To overcome these limitations, we leveraged our cohort of Angelman syndrome individuals and generated three pairs of iPSCs from patUPD15 cases and their corresponding familial controls. Since gene editing approaches for patUPD15 remain unfeasible, genetically matched patient-control pairs provide the most reliable model for this condition. To our knowledge, this is the most comprehensive set of iPSCs available for studying this molecular cause of Angelman syndrome.

At the molecular level, patUPD15 and imprinting defects caused by maternal PWS-IC loss of methylation differ from other Angelman syndrome cases due to the abnormal biallelic expression of several paternally expressed imprinted genes (*NDN*, *MAGEL2*, *MKRN3*, *SNRPN/SNURF*, *SNORD115*, *SNORD116*) alongside *UBE3A* loss. Our stem cell toolkit provides a unique opportunity to investigate the impact of UBE3A deficiency in a context where these neuronal genes are misregulated, helping to identify a distinct molecular signature of patUPD15 cases that could be targeted for future therapeutic strategies. This is particularly important given that patUPD15 (and imprinting defect) cases of Angelman syndrome were excluded from clinical trials (clinicaltrials.gov, NCT04259281, NCT04428281, NCT06914609, NCT06415344) using modified antisense oligonucleotide (mASO) technology to reinstate *UBE3A* expression by activating the paternal allele through RNase H-mediated degradation of the *UBE3A-ATS*. The exclusion stems from concerns that in patUPD15, this could lead to *UBE3A* overexpression due to activation of both alleles. This could be harmful, since *UBE3A* overexpression is linked to an autistic phenotype in individuals with maternal 15q duplication syndrome [24]. Our patUPD15 stem cell toolkit provides a unique humanized system to test ASOs targeting *UBE3A-ATS*, enabling the assessment of *UBE3A* expression levels and the potential benefits or risks of this promising treatment and other similar approaches [25–27]. This will provide crucial insights into the viability of therapies targeting paternal *UBE3A* activation for these patients.

By expanding the available models of Angelman syndrome to patUPD15 cases, our work contributes to a more comprehensive understanding of the disorder and paves the way for more targeted and effective therapeutic strategies for the different molecular causes of Angelman syndrome.

## Supplementary Information

Below is the link to the electronic supplementary material.

Supplementary file1 (PDF 32193 KB)

Supplementary file2 (XLSX 13 KB)

Supplementary file3 (XLSX 13 KB)

## Data Availability

The data that support the findings of this study are available on request from the corresponding author S.T.d.R.
