# Supplementary material for: A stem cell-based toolkit to model Angelman syndrome caused by paternal uniparental disomy of chromosome 15
Source: Hum Cell. 2025 Sep 16;38(6):161. doi: 10.1007/s13577-025-01287-8 (PMC12441060; doi:10.1007/s13577-025-01287-8)
Supplement: Supplementary file 1 — Supplementary file1 (PDF 32193 KB) [file 13577_2025_1287_MOESM1_ESM.pdf]

Fig. S1

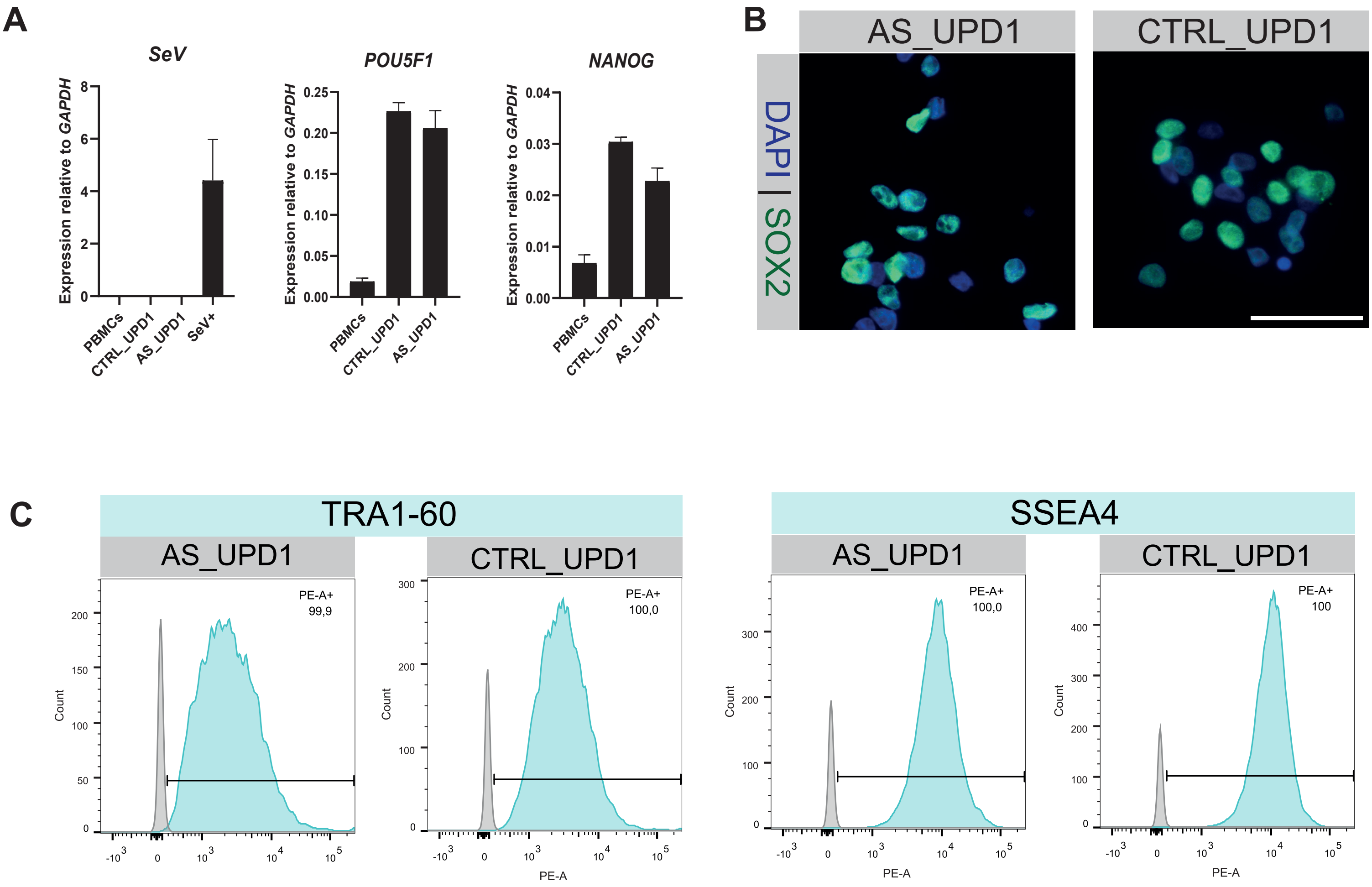

A
SeV
POU5F1
NANOG
Expression relative to GAPDH
PBMCs
CTRL_UPD1
AS_UPD1
SeV+
B
AS_UPD1
CTRL_UPD1
DAPI | SOX2
C
TRA1-60
SSEA4
AS_UPD1
CTRL_UPD1
PE-A+
99,9
100,0
100
Count
PE-A

Fig. S2

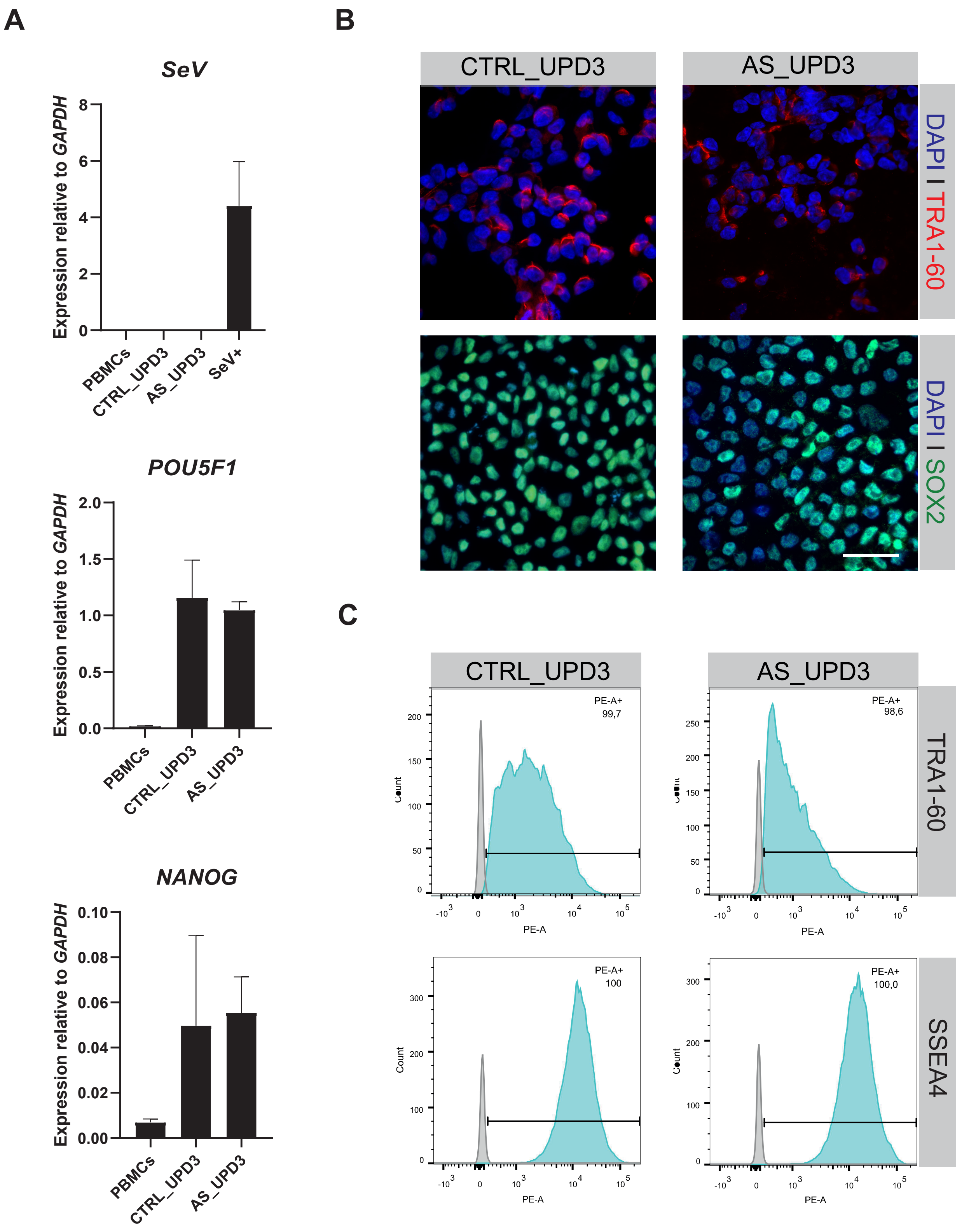
A
SeV
Expression relative to GAPDH
PBMCs
CTRL_UPD3
AS_UPD3
SeV+
POU5F1
NANOG
B
CTRL_UPD3
AS_UPD3
DAPI I TRA1-60
DAPI I SOX2
C
TRA1-60
SSEA4
PE-A+
99,7
98,6
100
100,0
Count
PE-A

Fig. S3

A

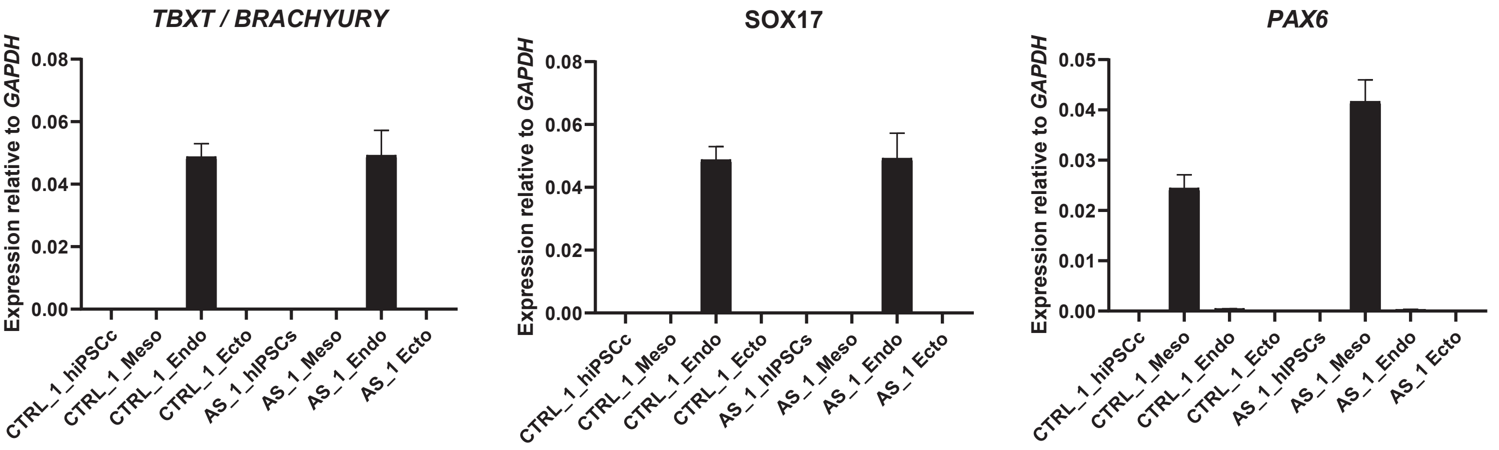
TBXT / BRACHYURY
SOX17
PAX6
Expression relative to GAPDH
0.08
0.06
0.04
0.02
0.00
0.05
0.03
0.01
CTRL_1_hiPSCc
CTRL_1_Meso
CTRL_1_Endo
CTRL_1_Ecto
AS_1_hIPSCs
AS_1_Meso
AS_1_Endo
AS_1 Ecto

B

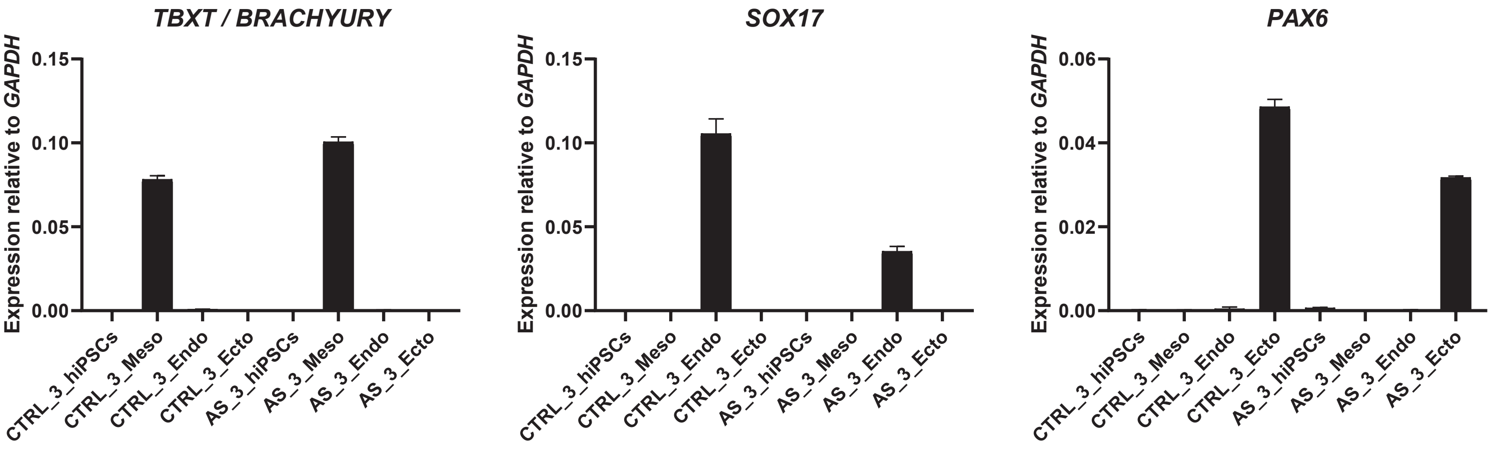
TBXT / BRACHYURY
SOX17
PAX6
Expression relative to GAPDH
0.15
0.10
0.05
0.00
0.06
0.04
0.02
CTRL_3_hiPSCs
CTRL_3_Meso
CTRL_3_Endo
CTRL_3_Ecto
AS_3_hiPSCs
AS_3_Meso
AS_3_Endo
AS_3_Ecto

Fig. S4

A

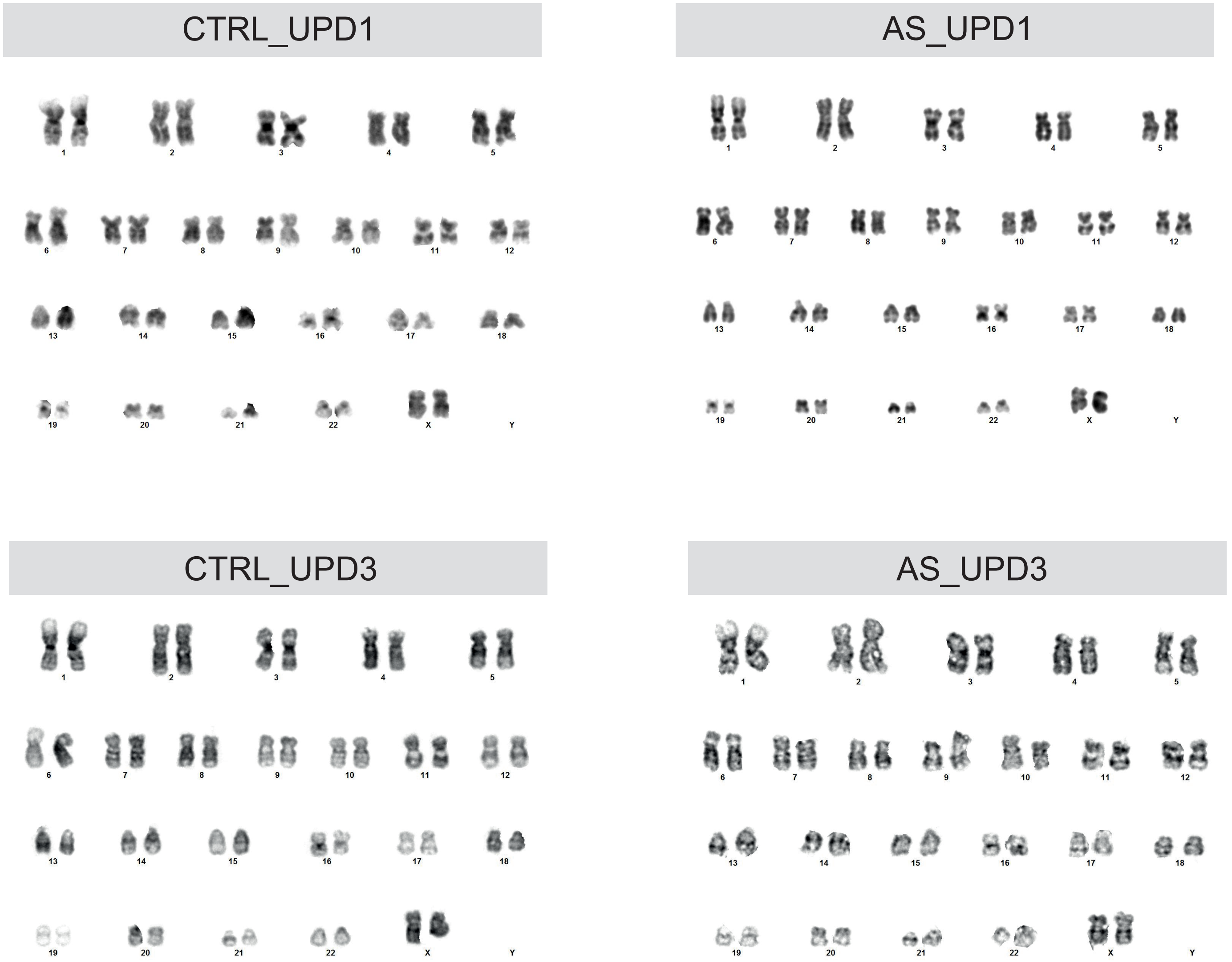
CTRL_UPD1
AS_UPD1
CTRL_UPD3
AS_UPD3

B

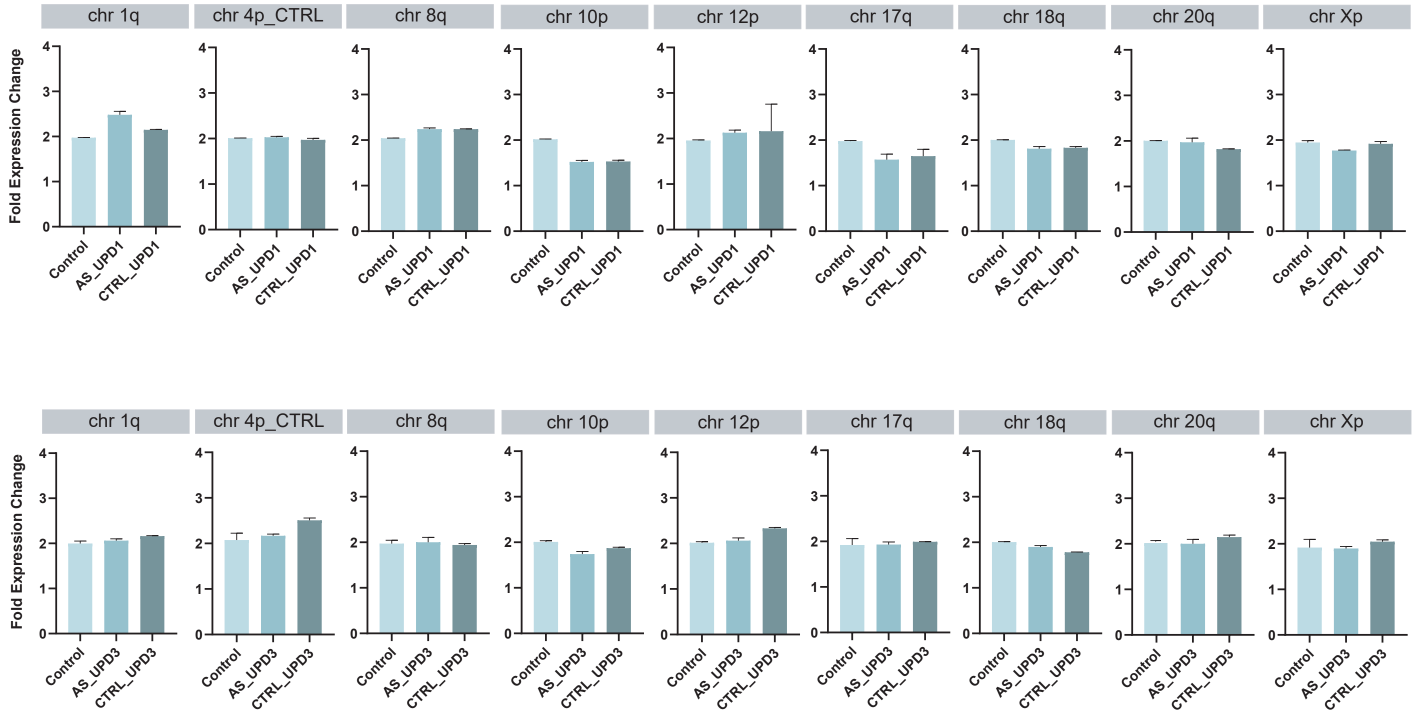
chr 1q
chr 4p_CTRL
chr 8q
chr 10p
chr 12p
chr 17q
chr 18q
chr 20q
chr Xp
Fold Expression Change
Control
AS_UPD1
CTRL_UPD1
AS_UPD3
CTRL_UPD3

Fig. S1 - Generation and characterization of stemness markers of newly generated CTRL-UPD1 and AS-UPD1 induced pluripotent stem cell lines.

A) Expression levels of SeV, POU5F1 and NANOG as measured by Reverse Transcription-quantitative Polymerase Chain Reaction (RT-qPCR) normalized for GAPDH housekeeping gene; On the left, the barplot shows SeV expression levels in PBMCs, CTRL_UPD1 and AS_UPD1 iPSC lines and infected samples at day 3 of reprogramming (SeV+); In the middle and on the right, the barplots show expression levels of POU5F1 and NANOG, respectively, in PBMCs, CTRL_UPD1 and AS_UPD1 iPSC lines; n=1 for all samples, barplots show mean +/- Standard Deviation (SD). **B)** Protein expression of stemness nuclear markers SOX2 in green by immunofluorescence, counterstained by DAPI in blue for both CTRL_UPD1 and AS_UPD1 iPSC lines; Scale bar: 50 µm **C)** Quantitative expression of the stemness membrane markers, TRA1-60 and SSEA4, in CTRL_UPD2 and AS_UPD2 iPSC lines by flow cytometry.

Fig. S2 - Generation and characterization of stemness markers of newly generated CTRL-UPD3 and AS-UPD3 induced pluripotent stem cell lines.

A) Expression levels of SeV, POU5F1 and NANOG as measured by Reverse Transcription-quantitative Polymerase Chain Reaction (RT-qPCR) normalized for GAPDH housekeeping gene; On the left, the barplot shows SeV expression levels in PBMCs, CTRL_UPD3 and AS_UPD3 iPSC lines and infected samples at day 3 of reprogramming (SeV+); In the middle and on the right, the barplots show expression levels of POU5F1 and NANOG, respectively, in PBMCs, CTRL_UPD3 and AS_UPD3 iPSC lines; n=1 for all samples, barplots show mean +/- SD. **B)** Protein expression of stemness markers TRA-1-60 in red (above) and SOX2 in green (below) by immunofluorescence, counterstained by DAPI in blue for both CTRL_UPD3 and AS_UPD3 iPSC lines; Scale bar: 50 µm. **C)** Quantitative expression of the stemness membrane markers, TRA1-60 and SSEA4, in CTRL_UPD3 and AS_UPD3 iPSC lines by flow cytometry.

Fig. S3 - Trilineage differentiation competency of newly generated CTRL UPD1, CTRL-UPD3 and AS-UPD1 and AS-UPD3 induced pluripotent stem cell lines.

A) Expression levels of TBXT/BRACHYURY, SOX17 and PAX6 markers as measured by Reverse Transcription-quantitative Polymerase Chain Reaction (RT-qPCR) normalized for GAPDH housekeeping gene in CTRL_UPD1 and AS_UPD1 induced pluripotent stem cells (iPSC) lines in the undifferentiated state and differentiated down to the mesoderm, endoderm and ectoderm lineages; n=1 for all samples, barplots show mean +/- SD. **B)** Expression levels of TBXT/BRACHYURY, SOX17 and PAX6 markers as measured by Reverse

Transcription-quantitative Polymerase Chain Reaction (RT-qPCR) normalized for GAPDH housekeeping gene in CTRL_UPD3 and AS_UPD3 iPSC lines in the undifferentiated state and differentiated down to the mesoderm, endoderm and ectoderm lineages; n=1 for all samples.

Fig. S4 – Genetic fidelity of newly generated CTRL UPD1, CTRL-UPD3 and AS-UPD1 and AS-UPD3 induced pluripotent stem cell lines.

A)Representative G-banding karyotype images of CTRL_UPD1 (46,XX)[31], AS UPD1 (46,XX)[25], CTRL-UPD3 (46,XX)[25] and AS-UPD3 (46,XX)[25] induced pluripotent stem cells. 300-500 band resolution. **B)** Copy number variations (CNVs) in specific chromosomal regions (1q, 8q, 10p, 12p, 17q, 20q11.21, Xp, and 18q) were determined by a qPCR-based genetic assay to screen for recurrent chromosomal abnormalities in iPSCs. Barplots show mean +/- SD. Statistical analysis was performed using one-way ANOVA with a pairwise test and no statistically significant abnormalities were detected below p-value of 0.05.
